# Oral glucocorticoids and incidence of hypertension in people with chronic inflammatory diseases: a population-based cohort study

**DOI:** 10.1503/cmaj.191012

**Published:** 2020-03-23

**Authors:** Teumzghi F. Mebrahtu, Ann W. Morgan, Robert M. West, Paul M. Stewart, Mar Pujades-Rodriguez

**Affiliations:** Leeds Institute of Biomedical and Clinical Sciences (Mebrahtu); Leeds Institute of Cardiovascular and Metabolic Medicine (Morgan), School of Medicine, University of Leeds; NIHR Biomedical Research Centre (Morgan, Stewart), Leeds Teaching Hospitals NHS Trust, Chapel Allerton Hospital; Leeds Institute of Health Sciences, (West, Pujades-Rodriguez), School of Medicine; Dean’s office, Faculty of Medicine & Health (Stewart), University of Leeds, Leeds, UK

## Abstract

**BACKGROUND:**

Only a few population-based studies have examined the association between glucocorticoids and hypertension, with inconsistent results. We aimed to investigate the effect of oral glucocorticoids on incidence of hypertension in adults with chronic inflammatory diseases.

**METHODS:**

We analyzed electronic health records from 389 practices in England during 1998–2017 of adults diagnosed with any of 6 chronic inflammatory diseases but with no previous diagnosis of hypertension. We used glucocorticoid prescription data to construct time-variant daily and cumulative variables of prednisolone-equivalent dose (cumulated from 1 year before the start of follow-up) and estimated incidence rates and adjusted hazard ratios (HRs) for hypertension using Cox regression analysis.

**RESULTS:**

Among 71 642 patients in the cohort, 24 896 (34.8%) developed hypertension during a median follow-up of 6.6 years. The incidence rate of hypertension was 46.7 (95% confidence interval [CI] 46.0–47.3) per 1000 person-years. Incidence rates increased with higher cumulative glucocorticoid prednisolone-equivalent dose, from 44.4 per 1000 person-years in periods of nonuse to 45.3 per 1000 person-years for periods with between > 0.0 and 959.9 mg (HR 1.14, 95% CI 1.09–1.19), to 49.3 per 1000 person-years for periods with 960–3054.9 mg (HR 1.20, 95% CI 1.14–1.27), and to 55.6 per 1000 person-years for periods with ≥ 3055 mg (HR 1.30, 95% CI 1.25–1.35). Cumulative effects were seen for the 6 diseases studied, but dose–response effects were not found for daily dose.

**INTERPRETATION:**

Cumulative dose of oral glucocorticoids was associated with increased incidence of hypertension, suggesting that blood pressure should be monitored closely in patients routinely treated with these drugs. Given that glucocorticoids are widely prescribed, the associated health burden could be high. **Trial registration:** ClinicalTrials. gov, no. NCT03760562.

Hypertension is a common preventable cause of cardiovascular morbidity and mortality and can substantially affect the quality of life and independence of older adults.^1^ Hypertension affects 1 in 5 of adults^2^ worldwide and is generally diagnosed and treated in primary health care services. The reported prevalence of hypertension among long-term users of glucocorticoids is more than 30%^3^ and it is found in 25%–93% of patients with Cushing syndrome. 4 The personal and economic impact of hypertension is likely to be higher among people with chronic systemic inflammatory diseases, who already have limitations to their activity. These diseases are often initially treated with oral glucocorticoids for a minimum of 3 months,^5^–^11^ with disease relapses and flares requiring additional glucocorticoid dose escalation in the following years.

It is widely reported that use of oral glucocorticoids is associated with an increased risk of hypertension and that this association is dose related.^3^,^12^ Evidence, however, remains inconsistent, and the pathophysiology of glucocorticoid-induced hypertension is unclear.^12^ Three previous population-matched case–control studies investigating glucocorticoid-related adverse events among patients with psoriasis^13^ or asthma^14^,^15^ reported conflicting results. These studies were of relatively small size (< 7000 patients) and had several limitations. For example, they excluded a substantial number of patients for different reasons (e.g., rheumatic diseases,^14^ < 30-day glucocorticoid exposure in 1 year^15^), considered only medication prescribed by specialists in outpatient or hospital services,^13^ and did not model risk in relation to changing glucocorticoid dose over time or reported unadjusted estimates.^15^ Moreover, none of these studies adjusted the estimates of dose–response by disease activity, which could also affect the risk of hypertension.

The primary aim of this research was to investigate the effect of oral glucocorticoid dose on incident hypertension in patients with 6 common chronic inflammatory diseases, using linked electronic health records in England. The study also examined whether adjustment for disease activity over time influenced the estimates.

## Methods

### Study design

This was a population-based record-linkage cohort study in England, using 3 data sources: Clinical Practice Research Datalink (CPRD)^16^ to identify diagnoses, laboratory test results and prescribed medication; Hospital Episode Statistics (www.hscic.gov.uk/hes) to identify diagnoses recorded during hospital admission; and the Office of National Statistics (www.ons.gov.uk/atoz?query=mortality&size=10) to obtain information on date of death and the index of multiple deprivation.^17^ The CPRD is widely used for research, and patients are representative of the United Kingdom population in terms of age, sex and ethnicity.^16^,^18^

### Study population

The study included all eligible patients registered in general practices of the CPRD who had consented to data linkage, between Jan. 1, 1998, and Mar. 15, 2017. We included patients when they were aged 18 years or older, had been registered in the general practice for 1 year or more, and had no previous hypertension (Appendix 1, Supplemental Figure 1, available at www.cmaj.ca/lookup/suppl/doi:10.1503/cmaj.191012/-/DC1). All had received a diagnosis of at least 1 of 6 chronic inflammatory diseases (inflammatory bowel disease, systemic lupus erythematosus, polymyalgia rheumatica, giant cell arteritis, rheumatoid arthritis and vasculitis) before or during the study period (codes are provided in Appendix 1, Supplemental Table 1). These diseases are commonly treated with glucocorticoids, but dose and treatment duration vary. In the UK, primary care physicians prescribe long-term glucocorticoids for these diseases even if guidance on treatment is provided by the specialist.

### Ethics approval

The study was approved by the Independent Scientific Advisory Committee for Medicines and Healthcare Products Regulatory Agency Database Research, reference 16_146.

### Definition of hypertension

We defined incident hypertension as the earliest date on which a diagnosis of hypertension, or ≥ 3 high systolic or diastolic blood pressure measures, was recorded within 12 months during follow-up. In sensitivity analysis, we also considered the date of first prescribed blood pressure–lowering medication during follow-up. Diagnosis codes for hypertension are shown in Appendix 1, Supplementary Table 2. We defined high systolic and diastolic blood pressure values as in current clinical guidelines: ≥ 140 mm Hg and ≥ 90 mm Hg, respectively.^1^ We defined history of hypertension at study entry (i.e., for exclusion from the study), considering diagnosis at any time and high blood pressure measurements or ≥ 1 prescription of blood pressure–lowering medication in the last year.

### Definition of oral glucocorticoid exposure

We defined glucocorticoid exposure status using all drug prescriptions for oral glucocorticoids issued in primary care. We extracted glucocorticoid doses from the directions given (e.g., once daily), product strength (e.g., 5 mg) and prescribed quantity. We converted dosages into prednisolone-equivalent dose to account for differences in potency of different types of glucocorticoids.^19^ See Appendix 1, Supplementary Table 3 for conversion rates.

To prevent length- and time-dependent bias,^20^ we analyzed periods covered or not by medication prescription (Appendix 1, Supplementary Methods).

We constructed time-variant continuous and categorical variables of daily dose and cumulative dose (which was cumulated from 1 year before the start of follow-up) (Appendix 1). The categories for daily dose were nonuse, > 0–4.9, 5.0–7.4 and ≥ 7.5 mg; and for cumulative dose were nonuse, > 0–959.9, 960–3, 054.9, and ≥ 3055 mg. We based cut-offs for the categorical variables on previous studies^21^–^23^ to facilitate comparability.

### Confounders

Our selection of confounders was guided by our clinical, biological and epidemiologic understanding. We selected confounding variables using DAGitty software.^24^ This approach for the analysis of causal inference in epidemiology determines covariate adjustment sets for minimizing confounding bias. Identified minimal sufficient adjustment sets are equally valid for causally different but statistically equivalent representations of the causal relationship examined, making it unnecessary to adjust for other covariates not included in that set. Of 10 robust minimal sufficient adjustment sets identified for estimating the causal effect of glucocorticoids on hypertension (Appendix 1, Supplemental Figure 3), we selected a set with age (continuous), index of multiple deprivation (5 categories), underlying inflammatory diseases (time-variant), non-oral glucocorticoids (binary), cardiovascular disease (binary), chronic renal disease stage 3 or 4 (binary) and scleroderma (time-variant).

### Statistical analysis

The start date of study follow-up was the earliest date on which all eligibility criteria were met. The end date of the follow-up was the last date of data collection in the general practitioner practice, practice deregistration or hypertension diagnosis date, whichever came first.

The end date of each drug prescription was not available, and we calculated it by adding the duration of days covered by the prescription to the prescription issue date. For each prescription recorded, we calculated its duration as the number of tablets prescribed divided by the daily dose. Where data on daily dose or the number of tablets prescribed were missing, we used truncated multiple imputation (Appendix 1, Supplemental Methods). We capped the maximum prescription duration at 90 days, which is the maximum recommended duration of prescribed medication in primary care. We then implemented a clinician-driven algorithm to improve the accuracy of imputed dose during tapering periods.

We calculated dose-specific incidence rates as the number of hypertension events divided by the total number of person-years at risk. We used Cox proportional hazard models to estimate risk of hypertension related to glucocorticoid dose. We tested the assumption of proportional hazard by allowing covariates to interact with analysis time. Where the assumption was violated, we included interaction terms with significant coefficients in the models.

We imputed missing data on daily doses and number of tablets prescribed using truncated regression and imputed 50 data sets (Appendix 1, Supplemental Methods). Information on outcome and covariates was complete, so no imputation was required. The association between prednisolone-equivalent dose and hypertension was nonlinear (*p* value for departure from linearity was < 0.001). We adopted 5% significance levels and 95% confidence intervals (CIs) throughout. We performed all analyses in STATA (version 14.1).

In sensitivity analyses, we investigated the effects of disease severity on dose-related risks of hypertension through adjustment by periods of disease activity and further adjusted estimates for hypertension-inducing medication use during follow-up. In addition, we estimated dose-related risks of hypertension for each chronic inflammatory disease and used mixed-effect models to account for variation at general practice level.

## Results

The study included 71 642 participants from 389 general practices. The mean age at study entry was 50.5 years (standard deviation [SD] 17.4). Sixty-two percent were women and 61 637 (86.0%) were white. The most common underlying condition was inflammatory bowel disease (25 162; 35.1%), followed by rheumatoid arthritis (20 214; 28.2%). Five percent of the patients (*n* = 3794) had a history of cardiovascular disease (Table 1), and 52 711 (73.6%) received hypertension-inducing medication during follow-up (Appendix 1, Supplemental Table 4).

**Table 1:** Patient baseline characteristics

Characteristic	No. (%) of patients* n = 71 642
Sociodemographic information	
Age, yr, mean ± SD	50.5 ± 17.4
Men	27 252 (38.0)
Ethnicity	
White	61 637 (86.0)
Black	776 (1.1)
Asian	2316 (3.2)
Index of multiple deprivation	
1st (least deprived)	13 139 (18.3)
5th (most deprived)	11 883 (16.6)
Body mass index in kg/m^2^, mean ± SD	25.7 ± 5.3
Chronic inflammatory disease†	
Giant cell arteritis	3219 (4.5)
Inflammatory bowel disease	25 162 (35.1)
Polymyalgia rheumatica	16 385 (22.9)
Rheumatoid arthritis	20 214 (28.2)
Systemic lupus erythematosus	3329 (4.7)
Vasculitis	4473 (6.2)
Comorbidities	
Cardiovascular diseases	3794 (5.3)
Stage 3 or 4 chronic kidney disease	797 (1.1)
Scleroderma	57 (0.2)
Non-oral glucocorticoids in last year	
Inhaled	5606 (7.8)
Nasal	3811 (5.3)
Intramuscular	297 (0.4)
Topical	1306 (1.8)
Rectal	3411 (4.8)

Note: SD = standard deviation.

* Unless stated otherwise.

† Figures include patients with prevalent and incident disease, and some patients could have more than 1 disease.

The average prescribed cumulative dose was 3204 mg prednisolone-equivalent dose. Patients with giant cell arteritis had the highest values (4410 mg prednisolone-equivalent dose), followed by patients with polymyalgia rheumatica (4364 mg prednisolone-equivalent dose). Overall, 40 648 (56.7%) patients had received a diagnosis of the underlying disease treated at study entry, while 30 994 (43.0%) had received the diagnosis, on average, 5 years before the study entry date (Appendix 1, Supplemental Table 5).

### Oral glucocorticoids and hypertension

In more than 532 994 person-years of follow-up, there were 24 896 (34.8%) incident cases of hypertension, 38 (0.2%) of them recorded as diagnoses of secondary hypertension, 18 140 (72.8%) as essential hypertension and 6718 (27.0%) ascertained through increases in blood pressure measurements (i.e., without recorded diagnosis). Median follow-up time was 6.5 years (interquartile range 2.7–11.8 yr) and the incidence rate was 46.7 (95% CI 46.0–47.3) per 1000 person-years. Incidence rates of hypertension increased with higher cumulative glucocorticoid doses and were higher during periods with prescribed glucocorticoids compared with periods of nonuse (Table 2).

**Table 2:** Observation time, overall incidence rates and time-variant oral glucocorticoids dose–related incidence rates of hypertension

Characteristic	Study population *n* = 71 642
Total person-years of follow-up	532 994
Total incident cases, *n* (%)	24 896 (34.8)
Time at risk per patient, median years (P25–P75)	6.5 (2.7–11.8)
Incidence rates per 1000 person-years (95% CI)	
Overall	46.7 (46.0–47.3)
Daily dose, mg	
Nonuse	46.1 (45.5–46.7)
> 0–4.9	53.6 (49.5–57.9)
5.0–7.4	52.8 (48.5–57.5)
≥ 7.5	50.7 (47.8–53.8)
Cumulative dose, mg*	
Nonuse	44.4 (43.7–45.4)
> 0–959.9	45.3 (43.5–47.2)
960–3054.9	49.3 (46.9–51.8)
≥ 3055	55.6 (54.0–57.2)

Note: CI = confidence interval, P25 = 25th centile, P75 = 75th centile.

* Time-variant cumulative dose since 1 year before study entry.

There was a significant increase in hazard ratio (HR) of hypertension for cumulative dose, but not for daily prednisolone-equivalent dose (Table 3). For example, the rates increased by 14% (HR 1.14, 95% CI 1.09–1.19), 20% (HR 1.20, 95% CI 1.14–1.27), and 30% (HR 1.30, 95% CI 1.25–1.35) when patients received between > 0.0 and 959.9 mg, 960 and 3054.9 mg, and ≥ 3055 mg cumulative prednisolone-equivalent dose over the follow-up period, respectively, compared with nonuse.

**Table 3:** Time-variant prescribed prednisolone-equivalent dose of oral glucocorticoids and risk of hypertension

Dose variable	HR (95% CI)*	HR (95% CI)†
Daily dose category, mg		
Nonuse (Ref.)	1	1
> 0–4.9	0.97 (0.90–1.06)	1.03 (0.95–1.11)
5.0–7.4	1.02 (0.93–1.11)	1.07 (0.99–1.17)
≥ 7.5	1.08 (1.01–1.14)	1.12 (1.06–1.19)
Cumulative dose, mg‡		
Nonuse (Ref.)	1	1
> 0–959.9	1.14 (1.09–1.19)	1.17 (1.12–1.22)
960–3054.9	1.20 (1.14–1.27)	1.24 (1.18–1.31)
≥ 3055	1.30 (1.25–1.35)	1.36 (1.31–1.41)

Note: CI = confidence interval, HR = hazard ratio, Ref. = reference category.

* Estimates adjusted for baseline age, index of multiple deprivation, non-oral glucocorticoid use (inhaled, nasal, intramuscular, intra-articular, topical or rectal), comorbidities (cardiovascular disease, chronic kidney disease and scleroderma) and type of chronic inflammatory disease.

† Estimates further adjusted for hypertension-inducing medication use during follow-up (drugs listed in Appendix 1, Supplemental Table 4).

‡ Time-variant cumulative dose since 1 year before study entry.

### Dose–response risk of hypertension by type of chronic inflammatory disease

**Figure 1:**
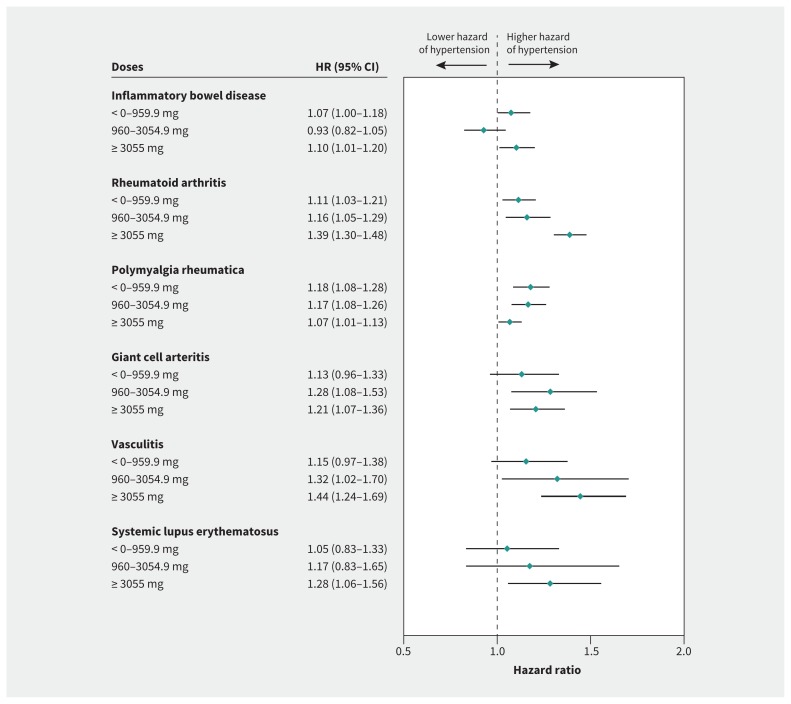
Time-variant cumulative prednisolone-equivalent dose of oral glucocorticoids and the risk of hypertension by type of chronic inflammatory disease. Note: estimates adjusted for age, index of multiple deprivation, non-oral glucocorticoids (inhaled, nasal, intramuscular, intra-articular, topical or rectal), comorbidities (cardiovascular disease, chronic kidney disease and scleroderma) and time-variant inflammatory chronic disease. CI = confidence interval, HR = hazard ratio.

**Figure 2:**
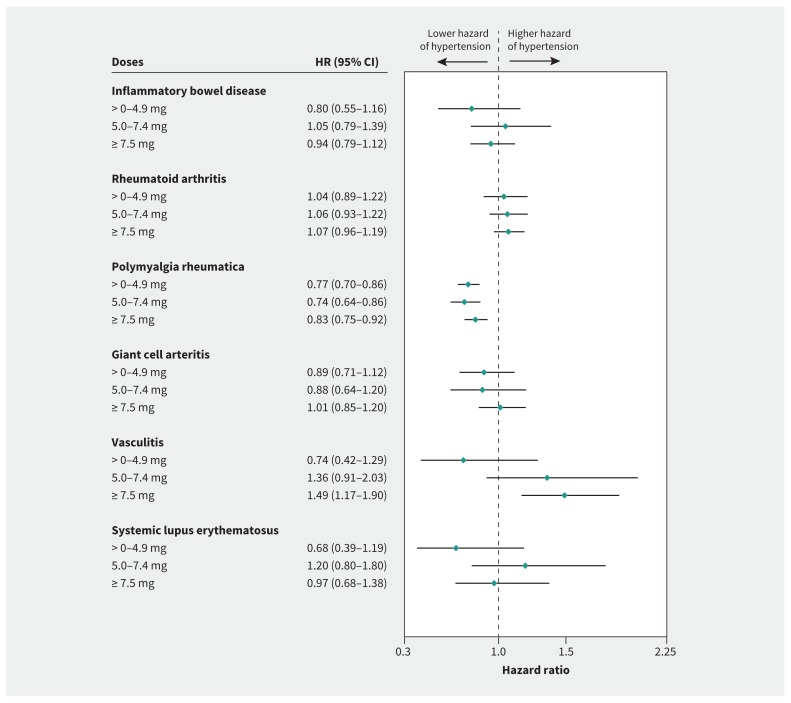
Time-variant daily prednisolone-equivalent dose of oral glucocorticoids and the risk of hypertension by type of chronic inflammatory disease. Note: estimates adjusted for age, index of multiple deprivation, non-oral glucocorticoid use (inhaled, nasal, intramuscular, intra-articular, topical or rectal), comorbidities (cardiovascular disease, chronic kidney disease and scleroderma) and time-variant chronic inflammatory disease. CI = confidence interval, HR = hazard ratio.

The hazard rate of hypertension increased with higher cumulative dose categories of oral glucocorticoids in all disease types except inflammatory bowel disease and polymyalgia rheumatica, in which the observed HRs were similarly increased for all cumulative dose levels (Figure 1). Evidence of dose–response for hypertension related to prescribed oral daily glucocorticoids was found only for vasculitis (HR 1.49, 95% CI 1.17–1.90 for a daily dose ≥ 7.5 mg; Figure 2). Protective effects were found for polymyalgia rheumatica (HR 0.83, 95% CI 0.75–0.92 for a daily dose ≥ 7.5 mg).

### Results from sensitivity analyses

In our sensitivity analyses, the estimates of dose–response for hypertension were generally similar to the primary analysis, but were higher for patients with systemic lupus erythematosus and polymyalgia rheumatica, when further adjusted for periods of disease severity or hypertension-inducing medication, or when mixed-effect models were used (Appendix 1, Supplemental Tables 6–8 and Table 3). Hazard ratios for all cumulative dose levels were similar when hypertension was defined considering blood pressure–lowering medication use during follow-up (*n* = 6468), but we found stronger dose–response relationships for daily and cumulative dose when these patients were excluded (Table 4).

**Table 4:** Sensitivity analyses of time-variant prescribed prednisolone-equivalent dose of oral glucocorticoids and the risk of hypertension

Dose variables	Inclusion of patients prescribed blood pressure–lowering medication (*n* = 6468) in the definition of incident hypertension, HR (95% CI)*	Exclusion of patients prescribed blood pressure–lowering medication (*n* = 6468) from analyses, HR (95% CI)*
Daily dose category, mg		
Nonuse (Ref.)	1	1
> 0–4.9	0.89 (0.82–0.97)	1.51 (1.39–1.63)
5.0–7.4	1.01 (0.93–1.10)	1.70 (1.56–1.85)
≥ 7.5	1.17 (1.11–1.24)	1.79 (1.68–1.90)
Overall cumulative dose category, mg		
Nonuse (Ref.)	1	1
> 0–959.9	1.17 (1.13–1.22)	1.31 (1.25–1.37)
960–3054.9	1.16 (1.11–1.22)	1.49 (1.41–1.58)
≥ 3055	1.20 (1.16–1.25)	1.85 (1.78–1.92)

Note: CI = confidence interval, HR = hazard ratio, Ref. = reference category.

* Models were adjusted for age, index of multiple deprivation, non-oral glucocorticoid use (inhaled, nasal, intramuscular, intra-articular, topical or rectal), comorbidities (cardiovascular disease, chronic kidney disease and scleroderma) and type of chronic inflammatory disease.

## Interpretation

In this large retrospective cohort study of 71 642 people with chronic inflammatory diseases, we found evidence of a dose–response in hypertension risk with higher cumulative doses of oral glucocorticoids. Specifically, when patients reached cumulative doses of between ≥ 0.0 and 959.9 mg, 960 and 3054.9 mg and ≥ 3055 mg prednisolone-equivalent dose during follow-up, the HR of hypertension increased by 14%, 20% and 30%, respectively, compared with nonuse. Estimates were similar, although higher for polymyalgia rheumatica and systemic lupus erythematosus, after adjusting for disease severity. In contrast, evidence of dose–response for prescribed daily dose was found only for vasculitis. The difference in effect between current daily and cumulative dose could be explained by delays in diagnosis of hypertension (i.e., more than 1 in 4 patients with incident hypertension had 3 or more measurements of high blood pressure but no recorded diagnosis of hypertension) or by a need for a higher dose or longer exposure to glucocorticoid therapy for hypertension to be clinically evident. This could also explain the inverse dose–response relationship found for polymyalgia rheumatica.

Although the link between endogenous glucocorticoid excess (Cushing syndrome) and hypertension is known,^4^,^25^ findings from previous risk association studies in patients with exogenous glucocorticoids have been inconsistent.^13^–^15^,^26^ Two matched case–control studies found evidence of increased risk of hypertension associated with oral glucocorticoids in patients with asthma^15^ or psoriasis.^13^ One study compared unadjusted prevalence of hypertension between patients with asthma without prescribed medication in the previous 2 years and patients with asthma with prescribed medication for ≥ 30 days per year (median of 1260 mg prednisolone-equivalent dose).^15^ The other study reported higher risk of hypertension in patients with newly diagnosed psoriasis who were prescribed a high cumulative glucocorticoid dose in the previous 6 months (≥ 45 defined daily dose) compared with nonusers in the last year (adjusted odds ratio [OR] 1.87, 95% CI 1.35–2.59).^13^ In contrast, a recent matched case–control study, based on the analysis of CPRD data from patients with asthma, reported no evidence of increased risk of hypertension for current (within 180 days), recent (180–365 days) and past use (> 365 days) of oral glucocorticoids, or evidence of dose–response for current daily or cumulative dose within the previous 180 days.^14^ Furthermore, Huscher and colleagues^26^ studied 779 patients with rheumatic arthritis who attended outpatient care services in Germany and found higher self-reported increases in blood pressure in the previous 6 months among patients with a daily dose > 7.5 mg prescribed glucocorticoids than among those prescribed lower doses (23.0% v. 18.8%).

Our study has certain strengths. We based the study on the analysis of electronic health records from patients registered in CPRD family practices. Patients included in CPRD have been shown to be representative of the UK population in terms of age, sex and ethnicity.^16^,^18^ The sample size was large, and errors in prescribed medication are minimal given the automatic recording of this information in CPRD. To account for the variation in potency of different oral glucocorticoids, doses were standardized (i.e., converted to prednisolone-equivalent dose). We prevented length- and time-dependent bias by analyzing the periods of nonexposure throughout the follow-up period. Furthermore, confounding variables were systematically selected using statistical tools (directed acyclic graphs) to minimize bias due to confounding and overadjustment.

### Limitations

The limitations of our study include the lack of recording of the end date of glucocorticoid prescriptions and the daily dose during tapering periods that we imputed using a combination of statistical methods and clinician-driven correction algorithms. We also underestimated glucocorticoid intake because of the lack of information on glucocorticoids administered or prescribed in hospital (e.g., high-dose glucocorticoid pulses in patients with inflammatory bowel disease, systemic lupus erythematosus or vasculitis). This, together with differences in administration patterns or regimens across diseases, might explain the difference in the dose–response relationship observed between inflammatory bowel disease and other diseases. Furthermore, information on oral glucocorticoid prescription was used as a proxy for the actual intake of the drugs that assumed optimal adherence.

We defined hypertension as a binary outcome, based on a recorded diagnosis, ≥ 3 high blood pressure values within 1 year or, in sensitivity analysis, use of blood pressure–lowering medication. However, we were unable to examine dose–response effects in relation to blood pressure measurements over time and the reversibility of drug effects. It was not possible to accurately determine which patients with systemic lupus erythematosus or vasculitis had a history of glomerulonephritis, which can lead to hypertension, but our analyses were adjusted for severe chronic kidney disease (stage 3 or 4).

### Conclusion

The findings of this study indicate that the effect of oral glucocorticoid cumulative dose on hypertension is substantial. We suggest that blood pressure be closely monitored for early identification and management of hypertension in patients with diseases treated with long-term glucocorticoids. Given that glucocorticoids are widely prescribed,^5^ the associated health burden could be high.
